# Outbreak detection in Harar town and Kersa district, Ethiopia using phylogenetic analysis and source attribution

**DOI:** 10.1186/s12879-024-09800-4

**Published:** 2024-08-26

**Authors:** Cecilie Thystrup, Tine Hald, Dinaol Belina, Tesfaye Gobena

**Affiliations:** 1 National Food Institute, Technical University of Denmark, Kgs. Lyngby, Denmark; 2 School of Biological Sciences and Biotechnology, Haramaya University, Dire Dawa, Ethiopia; 3 College of Veterinary Medicine, Haramaya University, Dire Dawa, Ethiopia; 4 School of Environmental Health Science, College of Health and Medical Sciences, Haramaya University, Dire Dawa, Ethiopia

**Keywords:** Surveillance, Outbreak detection, Low- and middle-income countries, Foodborne diseases, Diarrhea

## Abstract

**Background:**

Foodborne diseases (FBDs) represent a significant risk to public health, with nearly one in ten people falling ill every year globally. The large incidence of foodborne diseases in African low- and middle-income countries (LMIC) shows the immediate need for action, but there is still far to a robust and efficient outbreak detection system. The detection of outbreak heavily relies on clinical diagnosis, which are often delayed or ignored due to resource limitations and inadequate surveillance systems.

**Methods:**

In total, 68 samples of non-typhoidal *Salmonella* isolates from human, animal and environmental sources collected between November 2021 and January 2023 were analyzed using sequencing methods to infer phylogenetic relationships between the samples. A source attribution model using a machine-learning logit-boost that predicted the likely source of infection for 20 cases of human salmonellosis was also run and compared with the results of the cluster detection.

**Results:**

Three clusters of samples with close relation (SNP difference < 30) were identified as non-typhoidal *Salmonella* in Harar town and Kersa district, Ethiopia. These three clusters were comprised of isolates from different sources, including at least two human isolates. The isolates within each cluster showed identical serovar and sequence type (ST), with few exceptions in cluster 3. The close proximity of the samples suggested the occurrence of three potential outbreaks of non-typhoidal *Salmonella* in the region. The results of the source attribution model found that human cases of salmonellosis could primarily be attributed to bovine meat, which the results of the phylogenetic analysis corroborated.

**Conclusions:**

The findings of this study suggested the occurrence of three possible outbreaks of non-typhoidal *Salmonella* in eastern Ethiopia, emphasizing the importance of targeted intervention of food safety protocols in LMICs. It also highlighted the potential of integrated surveillance for detecting outbreak and identifying the most probable source. Source attribution models in combination with other epidemiological methods is recommended as part of a more robust and integrated surveillance system for foodborne diseases.

**Supplementary Information:**

The online version contains supplementary material available at 10.1186/s12879-024-09800-4.

## Introduction

Foodborne diseases (FBDs) represent a significant risk to public health, with nearly one in ten people falling ill every year worldwide [1]. Foodborne diseases typically spread through eating undercooked or raw foods, or through cross-contamination of food, hands, surfaces or equipment, but can also spread directly from animals-to-humans or person-to-person [2]. Meat, poultry and eggs are particularly high-risk foods, due to the risk of contamination during pre-processing and the prevalence of microbial agents in the animal reservoirs, like *Salmonella* spp. or other foodborne pathogens [3]. Non-typhoidal *Salmonella* can also spread through contaminated water, as well as the environment, where ill food handlers can contribute to the spread of disease [4, 5].

There are very limited data on the burden of foodborne disease in low- and middle-income countries (LMIC), which complicates the process of determining the magnitude of the problem, but recent numbers from 2019 estimates that diarrhea carries the third largest part of the burden of disease in Ethiopia with 2,898 disability-adjusted life years (DALYs) per 100,000 population [6]. While the full extent of FBDs in these regions remains underreported, the incidence of foodborne illnesses, particularly those related to *Salmonella* spp., indicates an urgent need for reliable outbreak detection systems [7, 8]. Several factors point to the high incidence in Africa, like poor hygiene, low or no food safety standards and rapid, unregulated use of antimicrobials [9]. The high incidence of foodborne diseases in African LMIC shows the immediate need for action, but there is still far to a robust and efficient outbreak detection and surveillance system [10]. The recognition of foodborne outbreaks is often a complex and challenging process in African LMIC, where countries face issues like lack of reporting, low infrastructural capacity, and poor address system [11, 12]. In Ethiopia, and other LMICs, evidence of the occurrence of FBD often relies on available outbreak data, which have been shown to be able to capture emerging risks, and serve as an effective first step towards the development and implementation of more comprehensive surveillance system long term [13]. However, the detection of outbreaks heavily relies on clinical diagnosis, which are often delayed or not performed due to resource limitations and inadequate technical capacity and reporting systems. If these outbreaks are detected, they can be used to determine the attribution of different sources, which, in turn, enables the quantification of cases attributable to specific pathogens [14].

The application of whole-genome sequencing (WGS) in outbreak detection is well-established and has shown a great potential for use in monitoring and establishing of transmissions routes of foodborne diseases [15]. While WGS does rely on some availability of relevant clinical isolates, it offers high-quality detection which can help to identify pathogens down to a strain level with high accuracy, while also being cost-effective. Linking isolates across different sources is based on the hypothesis of shared ancestry, where, if the isolates are genetically indistinguishable, it is indicated that the isolates stem from the same source. Genetical relatedness can be quantified in many ways, one of them being multilocus sequence typing (MLST), where the genetic variation in any of the loci in the household genes defines a different allele and informs the sequence type (ST) [16]. However, to draw reliable conclusions about the relatedness of the isolates, the genetic information should always be corroborated with epidemiological evidence [17]. Therefore, WGS can also be used in phylogenetic analysis to track the evolutionary relationship between strains, which can help in pinpointing the source of an outbreak [15].

There are also examples where the use of source attribution models has helped link sporadic human cases of specific illnesses to food and animal reservoirs [18]. With the use of genetic signatures from the genetic information (MLST, *k-*mers or resistance genes), these models are able to identify the most likely origin of the human infections, leading to a more comprehensive understanding of the transmission between humans, animals and food sources [19].

In this study, we investigated a collection of non-typhodial *Salmonella* isolates from humans, animals, food, and environmental sources collected in Harar region, Ethiopia from November 2021 to January 2023. The objective was to investigate the genetic relationship between the strains to make inferences about possible sources of human infections.

## Methods

### Data collection and laboratory analysis

The data was collected as part of a cross-sectional study in rural communities in Harar town and Kersa district of eastern Ethiopia between 2021 and 2023 as part of the “Foodborne Disease Epidemiology, Surveillance and Control in African Low- and Middle Income Countries” (FOCAL)-project. Fecal samples from children < 5 years of age who were admitted to health facilities with diarrhea, their caretakers and siblings were collected in a community-based quantitative and qualitative cross-sectional study. Samples from selected domestic animals, food products and environmental samples (water- and wastewater samples) were also collected from each household, from households that consented. Food products, including meat samples, and domestic animals were also sampled from markets and abattoirs that were directly or indirectly linked to cases. All samples were accompanied by relevant epidemiological data, including GPS coordinates of sampling.

Samples were collected in a sterile container and kept in a cold box containing ice packs before being transported to the microbiology laboratory of Haramaya University on the day of collection, and tested under 24 h after collection. The samples were processed using a Standard Operating Procedure (SOP) created for the FOCAL project, and identification of non-typhoid *Salmonella* was conducted using culture and biochemical tests. The isolates were then transported to the Kilimanjaro Clinical Research Institute (KCRI) – Biotechnology Laboratory for analysis by methods described in detail elsewhere [20, 21]. In total, 191 diarrheagenic bacterial isolates from 74 individual households, 24 markets and 33 abattoirs were obtained. All isolates were sequenced using an Illumina NextSeq 550 Sequencing Machine. Isolates of non-typhoid *Salmonella* was conducted using culture and biochemical tests, as described in [21].

### Bioinformatics analysis

All sequences were aligned against redundant databases using KMA [22] to determine the species of the isolate. A phylogenetic analysis was performed on the non-typhoidal *Salmonella* sequences to determine the relationship between the samples. We used the Call SNPs and Infer Phylogeny (CSI) phylogeny pipeline sequence [23] to find sequence variations shared between the different isolates based on single nucleotide polymorphisms (SNPs). FastTree was used to construct maximum likelihood phylogenetic trees based on these sequences variations in the genome shared between the isolates. The phylogenetic tree was annotated and visualized in iTOL [24].

Seven loci Multilocus sequence typing (7gMLST) were performed on the sequences using the pipeline developed by Center for Genomic Epidemiology [25] (Available at https://cge.food.dtu.dk/services/MLST/), which uses the scheme developed by Achtman et al. (2012) for the non-typhodial *Salmonella* sequences. The serotype of the isolates was determined using SeqSero [26] using the scheme developed by Kauffman-White. Due to the greater genetic diversity found in our samples and the lack of information regarding optimal SNP difference in an African context, samples with < 30 SNP difference were considered to be part of a group of closely related strains (cluster).

### Source attribution modelling

Source attribution was conducted in R (2023.03.0 + 386). We applied a machine learning algorithm developed by Munck et al. 2020 [19] to predict the source of infections in children with diarrhea. The model uses smaller pieces of the genomes, known as *k*-mers, to detect patterns in the genomes, using either a random forest (rf) classifier or a logit-boost (lb) classifier. When the model had been trained and achieved sufficient accuracy on predicting the true sources of sequences with known origin (i.e. those from potential sources), we used the model to predict the likely source of the human salmonellosis cases by including both the WGS sequences of non-typhodial *Salmonella* from the human cases and the sources.

The genomes were *k*-merized using KMC (version 3.0) [27] with different lengths of *k* (k = 7, 9 and 11) for increased accuracy. The frequency of the *k*-mers were combined into a matrix for all samples. To reduce the number of *k-*mers for the model, any *k-*mers with a standard deviation of 2 or less were dropped from the combined matrix.

Sources were grouped based on pathogen reservoirs to increase the accuracy of the model, under the assumption that beef originating from the cattle reservoir shared a large proportion of its non-typhoidal *Salmonella* strains with cattle.

Additionally, we used the Boruta-function from the Boruta-package [28] (version 8.0.0) and the caret package [29] (version 6.0–94) to reduce the number of features in the final model. The near-zero-variance algorithm was used to reduce the number of *k*-mers by removing the *k-*mers with low variance, and the Boruta-algorithm was used to select important features based on the random forest classifier.

Due to the imbalance in the number of samples obtained from each source, the data was up-sampled to the maximum number of samples obtained from a source. This was done to increase the accuracy of the model by including as many samples for training as possible.

Both the random forest and the logit-boost were evaluated. The sources were split into test- and training data, which were then used to randomly generate training data sets corresponding to 70% of the total number of samples. The remaining 30% were then used for K-fold cross-validation to evaluate the performance of the model. The accuracy for each model was assessed after ten iterations, and the model with the highest accuracy was selected for model construction. The model was subsequently applied to the human cases to estimate the probability of each case to originate from the specific sources. In the final prediction step, all original data from both human and sources were included, and no up-sampling step was performed.

## Results

### Phylogenetic analysis

**Fig. 1:**
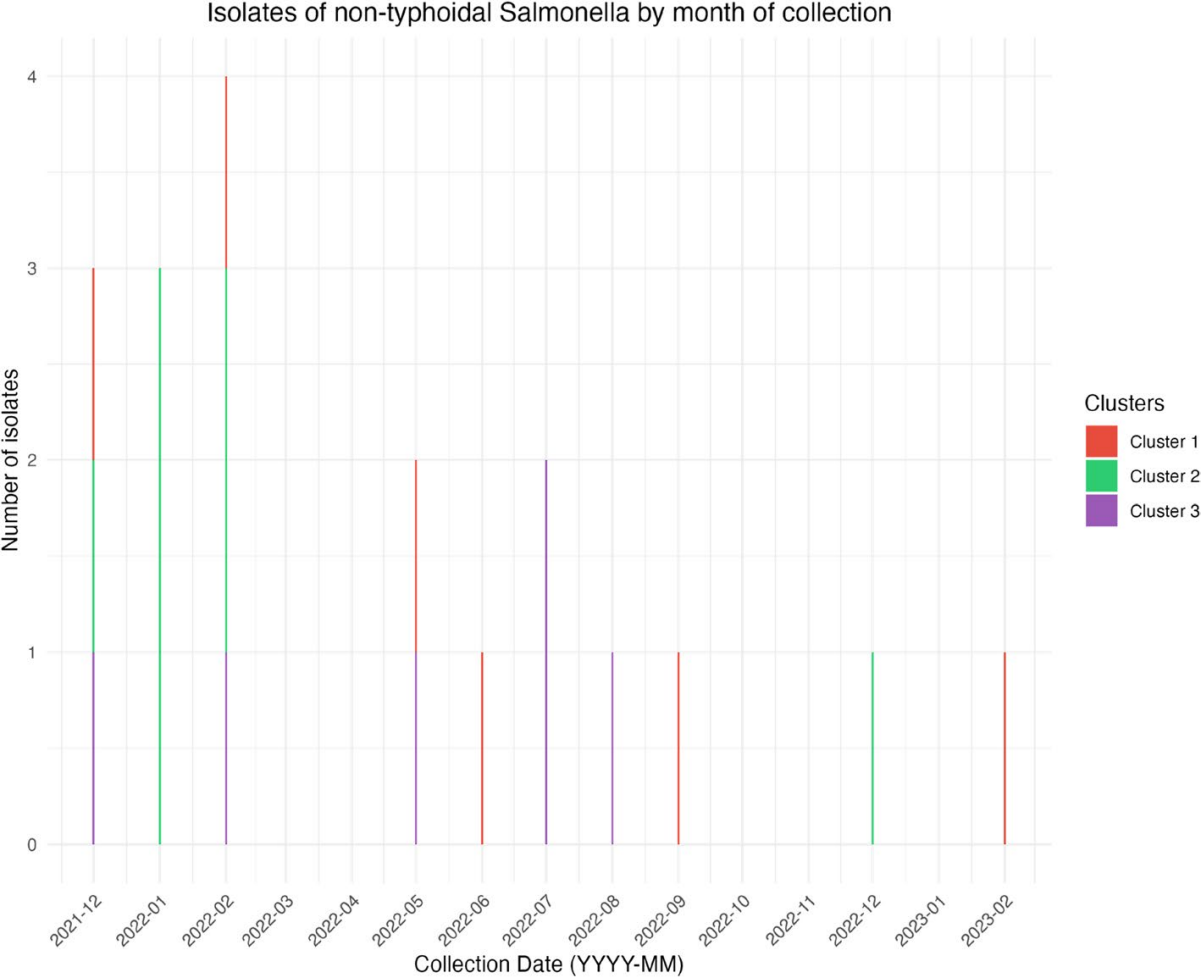
Epidemic curve of the isolates of non-typhoidal Salmonella collected between November 2021 and January 2023 (*n* = 68)

**Fig. 2:**
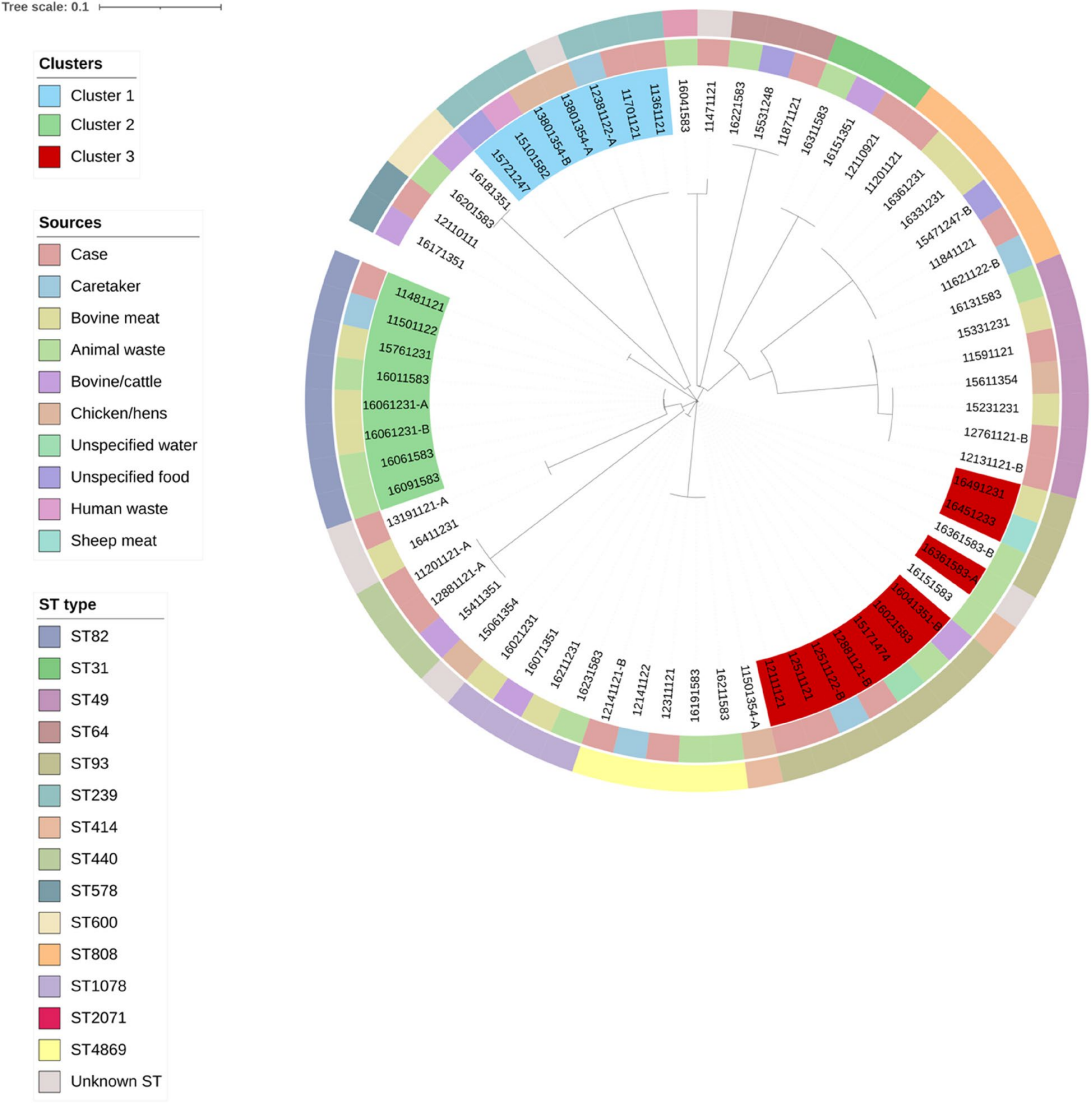
Phylogenetic tree showing all non-typhoidal Salmonella samples from Ethiopia (*n* = 68). Labels are colored according to the possible clusters found based on SNP difference (< 30). Inner circle denotes the source of the sample, and outer circle denotes the ST-type of the sample. Annotation was made in iTOL [24]

**Fig. 3:**
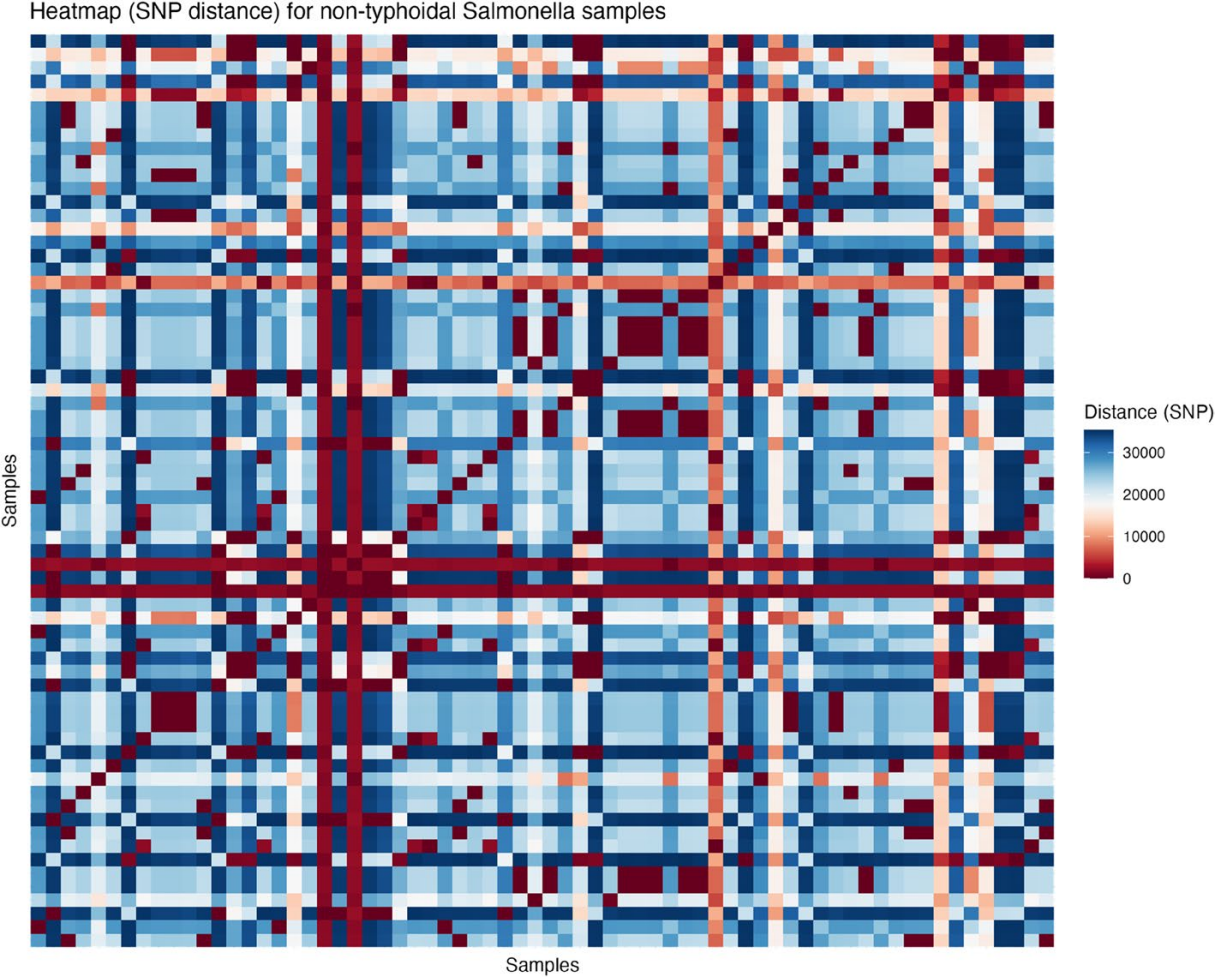
Heatmap showing the SNP distance between the non-typhoidal Salmonella samples (*n* = 68), ranging from 0 to 33,678

**Fig. 4:**
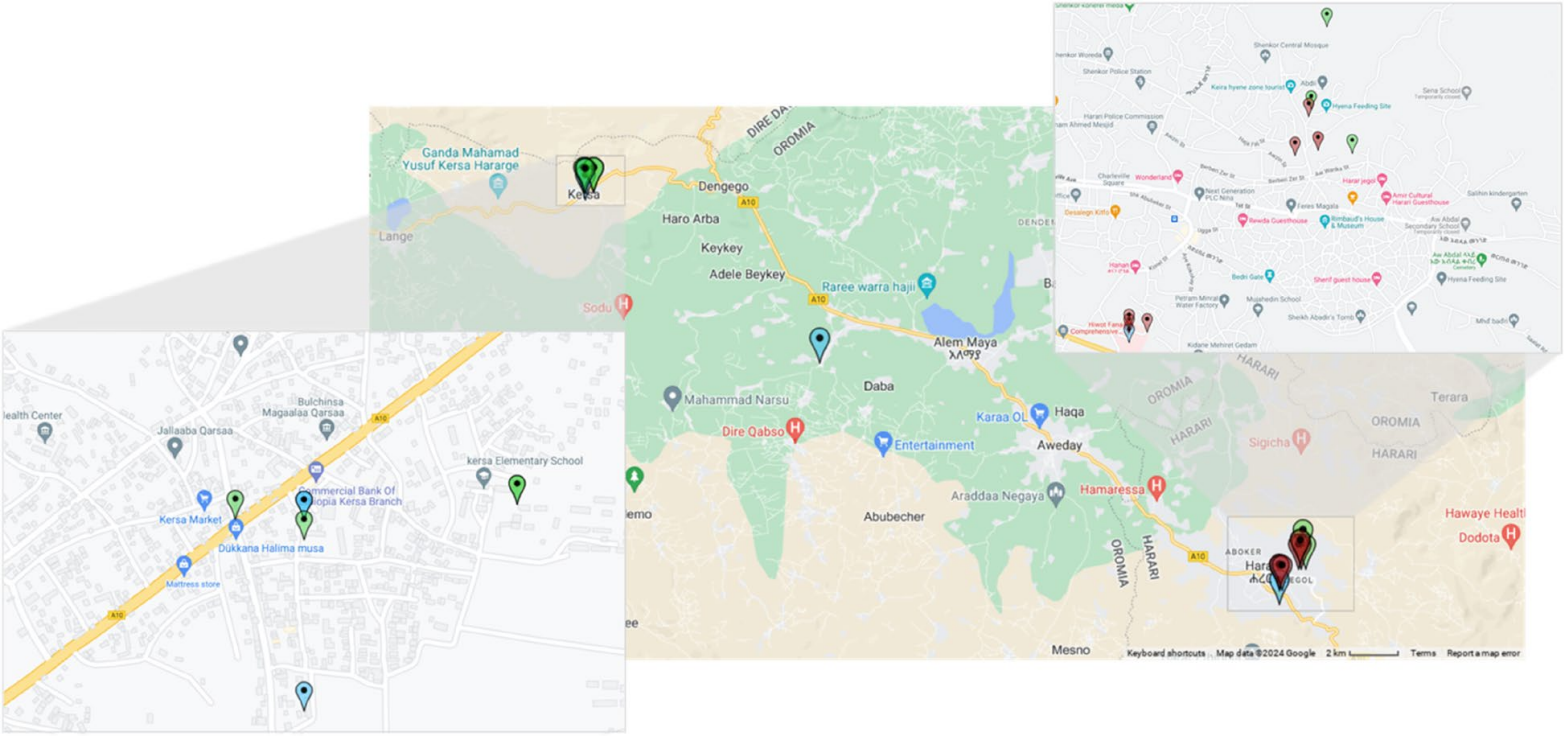
Map showing the locations of the clusters identified based on GPS coordinates (longitude, latitude). Blue icons denotes the location of the samples in cluster 1 (*n* = 6), green denotes samples in cluster 2 (*n* = 7) and red denotes samples in cluster 3 (*n* = 6). Map was created with Map Maker, using Google Maps API

Of the 191 isolates collected in the collection period from November 2021 to January 2023, 68 were identified as non-typhoidal *Salmonella*, where 20 came from children with diarrhea, five from caretakers, 17 from bovine meat or cattle, six from chicken or hens, 15 from animal waste, and two from non-dairy or non-meat food samples (Fig. 1). There were also a few singular isolates that came from sheep meat, human fecal matter (non-patient) and an unspecified water source*.* The species and ST were determined for all non-typhoidal *Salmonella* samples (Supplementary Table S1). The phylogenetic analysis revealed three possible clusters of non-typhoidal *Salmonella* in the samples (*n* = 20) (Fig. 2). A heatmap showing the SNP differences in the genomes showed that the samples ranged from having an SNP difference of 0 to 33,678, indicating that some samples were more closely related than others (Fig. 3). The three clusters identified showed identical sequence types (ST) and serovars (Table 1) in cluster 1 and 2, and very low SNP differences (0–3 for cluster 1, and 0–27 for cluster 2). Cluster 3 had a mix of STs and serovars, indicating that the isolates were not completely identical, despite the very low SNP difference (< 2). As a consequence, samples that did not have similar ST types were not considered to be part of cluster 3. The location of the samples identified revealed that the samples had been collected in mainly two areas around Kersa and Harar city, approximately 40 km apart (Fig. 4).

**Table 1:** Details of the samples identified in each cluster

Sample ID	Source of sample	Collection Date (DD-MM-YYYY)	Sequence type (ST)	Predicted serovar (Predicted antigenic profile)
**Cluster 1 (***n*** = 6)**				
15101582	Human waste (From Market)	05–05-2022	239	Gaminara (16:d:1,7)
13801354-A/B	Chicken/hens	03–10-2022	239	Gaminara (16:d:1,7)
12381122-A	Caretaker	03–06-2022	239	Gaminara (16:d:1,7)
15721247	Other food (from Market)	20–10-2022	239	Gaminara (16:d:1,7)
11701121	Case	26–02-2022	239	Gaminara (16:d:1,7)
11361121	Case	28–12-2021	239	Gaminara (16:d:1,7)
**Cluster 2 (***n*** = 7)**				
11481121	Case	10–01-2022	82	Muenchen (8:d:1,2)
11501122	Caretaker	12–01-2022	82	Muenchen (8:d:1,2)
15761231	Bovine meat (from Market)	27–12-2022	82	Muenchen (8:d:1,2)
16011583	Animal waste (from abattoir)	22–12-2021	82	Muenchen (8:d:1,2)
16061231-A/B	Bovine meat (from abattoir)	14–02-2022	82	Muenchen (8:d:1,2)
16061583	Animal waste (from abattoir)	24–01-2022	82	Muenchen (8:d:1,2)
16091583	Animal waste (from abattoir)	14–01-2022	82	Muenchen (8:d:1,2)
**Cluster 3 (***n*** = 6)**				
12111121	Case	17–05-2022	93	Eastbourne (9:e,h:1,5)
12511121	Case	07–07-2022	93	Eastbourne (9:e,h:1,5)
12511122-B	Caretaker	07–07-2022	93	Eastbourne (9:e,h:1,5)
16021583	Animal waste (from abattoir)	22–12-2021	93	18:e,h:1,5^a^
16041351-B	Bovine/cattle (from abattoir)	22–02-2022	93	Eastbourne (9:e,h:1,5)
16491231	Bovine meat (from abattoir)	28–08-2022	93	Eastbourne (9:e,h:1,5)

^a^No serovar name was reported for this sample

Cluster 1 contained six samples collected between December 28th 2021 and October 20th 2022 from children with diarrhea (*n* = 2), caretakers (*n* = 1), human waste from a market area (*n* = 1), household chicken (*n* = 1) and food sold at a market (*n* = 1). The phylogenetic analysis showed that all samples in this cluster carried ST239 and serovar Gaminara with antigenic profile 16:d:1,7, with a very low SNP difference (0) (Supplementary Table S2, Supplementary Figure S5), indicating that the *Salmonella* Gaminara isolates were highly related.

Cluster 2 contained seven samples collected between December 22nd 2021 and December 27th 2022 from children with diarrhea (*n* = 1), caretakers (*n* = 1), bovine meat sold at markets (*n* = 2) and animal waste from abattoirs (*n* = 3). All samples in this cluster were found to be of ST82 and serovar Muenchen with antigenic profile 8:d:1,2. The SNP difference in this cluster ranged from 0–27 SNPs, with the majority of the samples having a SNP difference of < 10, indicating that the samples in the cluster were closely related (Supplementary Table S3, Supplementary Figure S6).

Cluster 3 initially contained ten samples collected between December 22nd 2021 and August 30th 2022 from children with diarrhea (*n* = 3), caretakers (*n* = 1), animal waste from abattoirs and cattle from abattoirs (*n* = 2), an unknown water source from a market (*n* = 1), sheep (*n* = 2) and sheep/mutton meat (*n* = 1). The SNP difference in this cluster was very low (< 2) (Supplementary Table S4, Supplementary Figure S7), but the phylogenetic analysis showed that the samples carried different serovars and ST types (Supplementary Table S1), thereby indicating that not all the samples in the cluster were closely related. Selecting only the samples with similar ST type and serovar, we identified six samples collected between February 22nd 2022 and August 28th 2022 from children with diarrhea (*n* = 2), caretakers (*n* = 1), bovine meat (*n* = 1), cattle (*n* = 1) and animal waste (*n* = 1) from different markets. These samples all had ST93 and, except for one sample, carried serovar Eastbourne with antigenic profile 9:e,h:1,5. The sample that did not carry the Eastbourne serovar had antigenic profile 18:e,h:1,5. This indicated that these samples were closely related.

### Source attribution

The model used random forest and logit-boost to predict the likely source of the 20 salmonellosis cases based on the matrix containing 158,759 11-mers from five different sources (Table 2). The feature reduction found 3 important features after feature reduction, which were included in the model construction. Due to the imbalanced sampling of the different sources, the model selection was run with both the baseline data and the up-sampled data and the accuracy of both were compared (Table 3).

**Table 2:** Number of non-typhoidal Salmonella samples from each source (*n* = 64) before and after up-sampling

	Bovine/cattle	Caretaker	Chicken/hens	Waste (animal)	Waste (human)	Cases
Baseline data	17	5	6	15	1	20
Up-sampled data	17	17	17	17	17	17

**Table 3:** Average accuracy and 95% confidence interval (CI) for the baseline dataset and up-sampled dataset

	Model selection	
	Average accuracy (95% CI)	
	Random forest (rf)	Logit-boost (lb)
Baseline	0.393 (0.299–0.487)	0.472 (0.448–0.496)
Up-sampled data	0.658 (0.616–0.699)	0.716 (0.661–0.770)

The model was evaluated based on the average accuracy obtained from 10 iterations of sevenfold cross-validation. Average accuracy was highest for the logit-boost-model for the up-sampled data (0.716, 95% CI: 0.661–0.770), and thus this model was used for model construction.

**Fig. 5:**
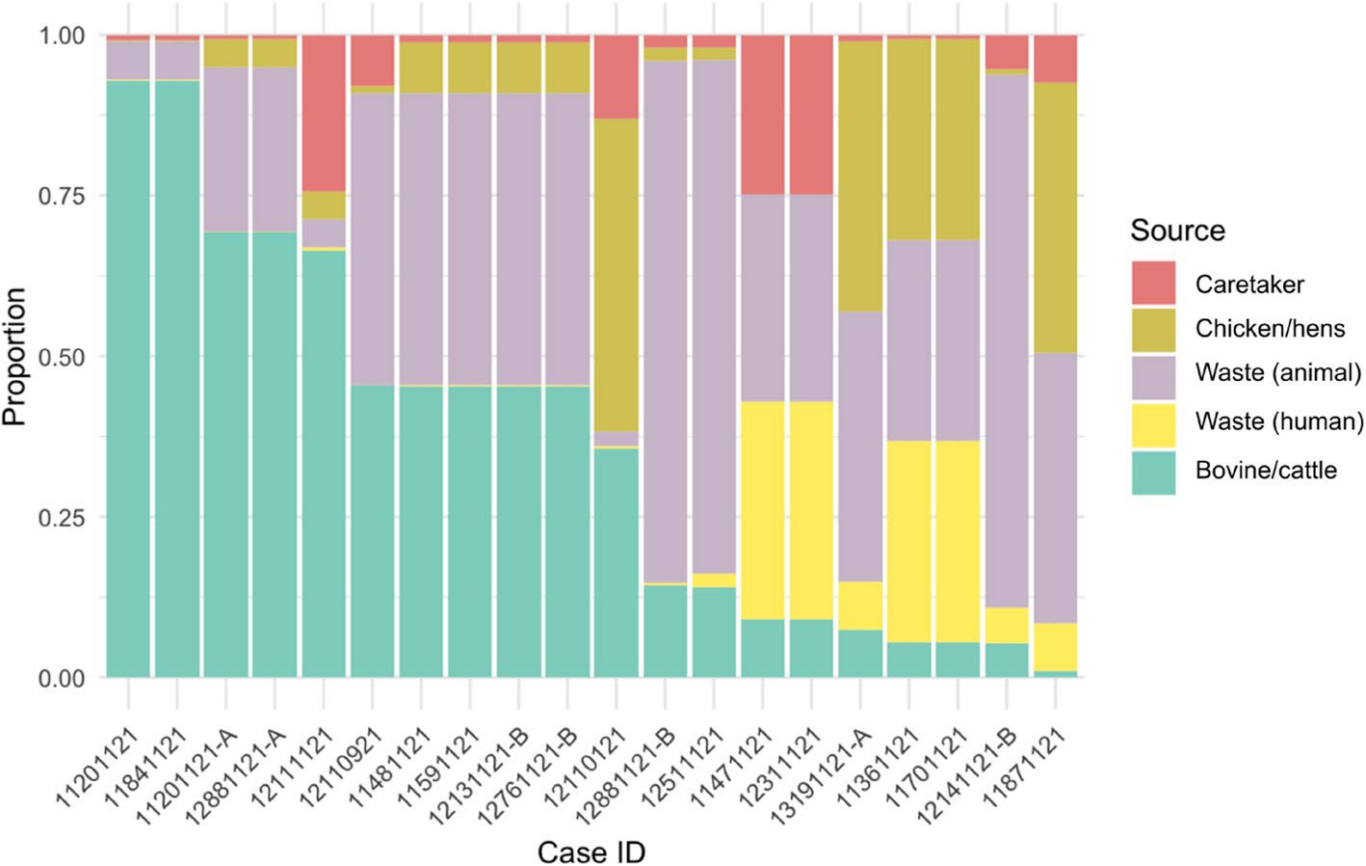
Prediction of human cases of salmonellosis from the machine learning model (*n* = 20). Each bar corresponds to one human case, with the course-specific likelihoods for each case cumulatively represented on the y-axis

The logit-boost model predicted 100% of the human salmonellosis cases (Table 4), with a kappa value of 0.554 and a valid accuracy of 0.64 (95% CI 0.425–0.82). The model predicted the main source of infection to be from either animal waste or bovine/cattle (15/20), with few exceptions (Table 4). The probability of each case originating from a specific source also varied between the cases (Fig. 5).

**Table 4:** Number of human salmonellosis cases attributed to each source (*n* = 20)

Bovine/cattle	Caretaker	Chicken/hens	Waste (animal)	Waste (human)
*7*	1	2	8	2

## Discussion

The primary objective of our study was to investigate the genetic relationship between the strains to make inferences about possible sources of human infections, using samples collected in the Harar region of Ethiopia. A total of 68 non-typhoidal *Salmonella* isolates were identified, which were assessed using SNP cluster analysis and phylogenetic analysis. The species was determined for 20 of the isolates, which included Gaminara, Muenchen, and Eastbourne. By systematically collecting samples over an extended period, it was possible to identify and analyze clusters of specific pathogens, thereby highlighting the usefulness of an integrated surveillance system where samples are systematically collected and investigated for potential bacterial infections. The identification of three distinct clusters involving human, animal, food, and environmental samples suggested the occurrence of three separate outbreaks of non-typhoidal *Salmonella* in the period between December 2021 and December 2022. The utilization of a source attribution model enabled us to infer the probable origins of human salmonellosis cases in the region. Aligning these predictions with the outbreaks we detected pointed to meat, particularly beef and chicken, sold in local markets and abattoirs as potential sources of the outbreaks. This approach highlights the occurrence and spread of non-typhoidal *Salmonella* in rural communities and emphasizes the advantages of having a surveillance system that integrates primary health data like data from diarrheagenic children and non-clinical isolates from potential sources such as food and animals.

We found that non-typhoidal *Salmonella* isolates with minimal genetic variation, specifically zero or one SNP, were present in all of the clusters, collected over several months. This observation suggested that all isolates had some sort of close relationship, based on their low mutation rate [30]. Unlike other studies, which have found cut-off values for SNP outbreak cluster detection of non-typhoidal *Salmonella* ranging between 0 and 10 SNPs [31–33], our study uses a broader threshold of up to 30 SNPs. This decision accounts for the greater genetic diversity we found in our samples, as well as the lack of information regarding SNP differences in African contexts. For that reason, we deemed it reasonable to increase the cutoff for outbreak detection, if the results were supported by results from in silico serotyping. Nonetheless, two of the identified clusters showed very little genetic variation (< 3 SNP differences), which further strengthened the relatedness of these isolates.

The samples in cluster 3 showed close genetic similarity with a SNP difference < 2, where all but one sample had the same predicted antigen profile. This discrepancy might be attributed to horizontal gene transfer mechanisms that allow for genetic recombination or the acquisition of new genes, altering the surface antigens of the pathogen [34]. However, the variance in the antigen profile could also be explained by the fact that the antigen profile of the serovar is predicted rather than confirmed, which could lead to inaccuracies in the serovar identification, especially if subtle genetic changes affect antigen expression [35]. Further experimental validation and epidemiological data would be needed to clarify these findings.

The genetic relatedness of the non-typhoidal *Salmonella* isolates within each cluster and the fact that the isolated were recovered in close geographical proximity, indicated three potential outbreaks of non-typhoidal *Salmonella.* Other studies have confirmed that all three implicated serovars have been found previously in African outbreaks, corroborating the notion that they could be linked to recent or ongoing outbreaks within the region [36–38].

This study provides valuable insights into the transmission of non-typhoidal *Salmonella*, but is subject to limitations that must be considered. The low sample size of the study, emphasized by the low number of samples in the detected clusters, may affect the generalizability of the findings. Additionally, despite the longitudinal collection of samples across several years, temporal constraints may still limit the applicability of the results over different time periods and geographical areas, especially when taking into account that Ethiopia is a geographically diverse country. Despite this, we were able to determine that bovine or cattle appears to be important sources of human salmonellosis in Ethiopia. The source attribution model showed varying levels of certainty, where some cases exhibited a more balanced probability distribution than others. This indicated that some cases of human salmonellosis were more difficult to attribute than others, which may be explained by the sources of *Salmonella* being diverse, making it challenging to attribute the infection to a single point of origin. Factors such as the widespread presence of *Salmonella* in many animal reservoirs as well as cross-contamination between food products could make the transmission routes more difficult to predict, which highlights the limitations in our study. Additionally, differences in the patient’s dietary habits or their contact with animals and contaminated environments could lead to some cases being predicted with low certainty to multiple sources, due to multiple exposures. Finally, the low sample size may not be able to capture the true genetic diversity in *Salmonella*, which also introduce further uncertainty into the attribution estimates.

The results from the source attribution indicated primarily animal reservoirs, bovine in particular, as the cause of the majority of the human salmonellosis cases. However, as the majority of the samples included in the model came from bovine sources, it is important to consider that the model might be skewed towards this source. To address this in the future, studies should aim for a more balanced data sampling approach across different sources.

Nonetheless, combining the findings from the source attribution model and the outbreak detection suggest that interventions aimed at reducing non-typhoidal *Salmonella* transmission should focus on these food-producing animals and their products, improving hygiene and handling practices in the slaughterhouses and markets.

This study provides evidence that WGS is a powerful tool for detecting and investigating outbreaks in Africa, despite the temporal width of the sample collection. However, the utility of WGS is contingent upon the availability of relevant and representative samples, which requires resources to obtain, analyse and report. In Ethiopia, integrated disease surveillance and response (IDSR) system is used for detecting sporadic cases and planning for responses of infectious diseases in the country [39]. The implementation of IDSR within the healthcare system is essential due to poor practices of health professionals, weak infrastructure, and inadequate surveillance processes [40, 41]. Non-typhoidal *Salmonella* is among the leading foodborne pathogens in the country, and although illnesses caused by this pathogen represent a large public health challenge, the full scope of the issue remains unclear due to underdiagnosis and a general tendency of the population to underutilize health services [39]. Furthermore, the community’s practice of consuming raw meat, along with the potential for non-typhoidal *Salmonella* to contaminate other uncooked foods, may intensify this issue. The existence of non-typhoidal *Salmonella* in beef could be regarded as a potential source of contamination in humans and could be the main risk factor for outbreaks in the community [42, 43].

## Conclusions

The findings of this study revealed the potential of three outbreaks of non-typhoidal *Salmonella* occurring in eastern Ethiopia, emphasizing the importance of targeted intervention of food safety protocols in LMIC. It also showed the potential of using WGS and source attribution models to predict potential sources of infection, which in turn could support public health officials and policy makers in their decisions to implement food safety protocols to mitigate the risk of *Salmonella* infections in rural communities in LMIC.

### Supplementary Information

Supplementary Material 1.

## Data Availability

The data that supports the findings of this study are available in the European Nucleotide Archive (ENA) under accession number PRJEB73590 or upon reasonable request from authors.

## Abbreviations

FBD: Foodborne disease
LMIC: Low- and Middle-Income Countries
DALY: Disability-adjusted life year(s)
WGS: Whole-Genome Sequencing
MLST: Multi-Locus Sequence Typing
FOCAL: Foodborne Disease Epidemiology, Surveillance and Control in African Low- and Middle Income Countries
ST: Sequence Type
SOP: Standard Operating Procedure
SNP: Single-Nucleotide Polymorphism
CI: Confidence Interval
IDSR: Integrated Disease Surveillance and Response
