# Supplementary material for: Outbreak detection in Harar town and Kersa district, Ethiopia using phylogenetic analysis and source attribution
Source: BMC Infect Dis. 2024 Aug 26;24:864. doi: 10.1186/s12879-024-09800-4 (PMC11348558; doi:10.1186/s12879-024-09800-4)

Supplementary Material

Outbreak detection in Harar town and Kersa District, Ethiopia using Phylogenetic analysis and Source Attribution

Thystrup et al

**Supplementary Table S1:** Sequence types (STs) and serovars determined for all *S. enterica* isolates included in the analysis (n=68)

| Sample ID | ST Type | Predicted antigenic profile | Predicted serotype |
| --- | --- | --- | --- |
| 11201121 | 808 | 8:e,h:1,5 | Kottbus |
| 11361121 | 239 | 16:d:1,7 | Gaminara |
| 11471121 | Unknown ST | 4:r:1,2 | Heidelberg |
| 11481121 | 82 | 8:d:1,2 | Muenchen |
| 11501122 | 82 | 8:d:1,2 | Muenchen |
| 11591121 | 49 | 4:e,h:1,2 | Saintpaul |
| 11701121 | 239 | 16:d:1,7 | Gaminara |
| 11841121 | 808 | 8:e,h:1,5 | Kottbus |
| 11871121 | 64 | 3,10:e,h:1,6 | Anatum |
| 12110111 | 578 | 18:f,g:- | I 18:f,g:- |
| 12110921 | 31 | 8:e,h:1,2 | Newport |
| 12111121 | 93 | 9:e,h:1,5 | Eastbourne |
| 12141122 | 4869 | 3,10:e,h:1,2 | Vejle |
| 12311121 | 4869 | 3,10:e,h:1,2 | Vejle |
| 12511121 | 93 | 9:e,h:1,5 | Eastbourne |
| 15061354 | Unknown ST | 18:z4,z23:- | Cerro |
| 15101582 | 239 | 16:d:1,7 | Gaminara |
| 15171474 | 93 | 9,46:e,h:1,5 | Waedenswil |
| 15231231 | 49 | 4:z10:1,2 | Haifa |
| 15331231 | 49 | 4:e,h:1,2 | Saintpaul |
| 15411351 | 440 | 35:f,g:- | Adelaide |
| 15531248 | 64 | 3,10:e,h:1,6 | Anatum |
| 15611354 | 49 | 4:z10:1,2 | Haifa |
| 15721247 | 239 | 16:d:1,7 | Gaminara |
| 15761231 | 82 | 8:d:1,2 | Muenchen |
| 16011583 | 82 | 8:d:1,2 | Muenchen |
| 16021231 | 1078 | 18:z4,z23:- | Cerro |
| 16021583 | 93 | 18:e,h:1,5 | I 18:e,h:1,5 |
| 16041583 | 2071 | 4:r:1,2 | Heidelberg |
| 16061583 | 82 | 8:d:1,2 | Muenchen |
| 16071351 | 1078 | 18:z4,z23:- | Cerro |
| 16091583 | 82 | 8:d:1,2 | Muenchen |
| 16131583 | 49 | 4:e,h:1,2 | Saintpaul |
| 16151351 | 31 | 8:e,h:1,2 | Newport |
| 16151583 | 414 | 9:e,h:1,5 | Eastbourne |
| 16171351 | 578 | 13:f,g:- | Havana |
| 16181351 | 600 | 3,10:b:1,5 | Butantan |
| 16191583 | 4869 | 3,10:e,h:1,2 | Vejle |
| 16201583 | 600 | 3,10:b:1,5 | Butantan |
| 16211231 | 1078 | 18:z4,z23:- | Cerro |
| 16211583 | 4869 | 3,10:e,h:1,2 | Vejle |
| 16221583 | 64 | 3,10:e,h:1,6 | Anatum |
| 16231583 | 1078 | 18:z4,z23:- | Cerro |
| 16311583 | 31 | 8:e,h:1,2 | Newport |
| 16331231 | 808 | 8:e,h:1,5 | Kottbus |
| 16361231 | 808 | 8:e,h:1,5 | Kottbus |
| 16411231 | Unknown ST | 8:l,v:1,2 | Litchfield |
| 16451233 | 93 | 3,10:e,h:1,5 | Muenster |
| 16491231 | 93 | 9:e,h:1,5 | Eastbourne |
| 11201121-A | 440 | 35:f,g:- | Adelaide |
| 11501354-A | 414 | 9:e,h:1,5 | Eastbourne |
| 11621122-B | 808 | 8:e,h:1,5 | Kottbus |
| 12131121-B | 49 | 4:z10:1,2 | Haifa |
| 12141121-B | 4869 | 3,10:e,h:1,2 | Vejle |
| 12381122-A | 239 | 16:d:1,7 | Gaminara |
| 12511122-B | 93 | 9:e,h:1,5 | Eastbourne |
| 12761121-B | 49 | 4:z10:1,2 | Haifa |
| 12881121-A | 440 | 35:f,g:- | Adelaide |
| 12881121-B | 93 | 3,10:e,h:1,5 | Muenster |
| 13191121-A | Unknown ST | 3,10:l,v:1,6 | London |
| 13801354-A | Unknown ST | 16:d:1,7 | Gaminara |
| 13801354-B | 239 | 16:d:1,7 | Gaminara |
| 15471247-B | 808 | 8:e,h:1,5 | Kottbus |
| 16041351-B | 93 | 9:e,h:1,5 | Eastbourne |
| 16061231-A | 82 | 8:d:1,2 | Muenchen |
| 16061231-B | 82 | 8:d:1,2 | Muenchen |
| 16361583-A | Unknown ST | 3,10:e,h:1,2 | Vejle |
| 16361583-B | 93 | 9,46:e,h:1,5 | Waedenswil |

**Supplementary Table S2:** SNP difference matrix for samples in cluster 1

|  | 15101582 | 13801354-B | 12381122-A | 15721247 | 11701121 | 11361121 |
| --- | --- | --- | --- | --- | --- | --- |
| **15101582** | 0 | 0 | 0 | 0 | 0 | 0 |
| **13801354-B** | 0 | 0 | 0 | 0 | 0 | 0 |
| **12381122-A** | 0 | 0 | 0 | 0 | 0 | 0 |
| **15721247** | 0 | 0 | 0 | 0 | 0 | 0 |
| **11701121** | 0 | 0 | 0 | 0 | 0 | 0 |
| **11361121** | 0 | 0 | 0 | 0 | 0 | 0 |

**Supplementary Table S3**: SNP difference matrix for samples in cluster 2

|  | 11481121 | 11501122 | 15761231 | 16011583 | 16061231-A/B | 16061563 | 16091583 |
| --- | --- | --- | --- | --- | --- | --- | --- |
| **11481121** | 0 | 0 | 1 | 27 | 1 | 24 | 1 |
| **11501122** | 0 | 0 | 1 | 27 | 1 | 24 | 1 |
| **15761231** | 1 | 1 | 0 | 26 | 0 | 23 | 0 |
| **16011583** | 27 | 27 | 26 | 0 | 26 | 3 | 26 |
| **16061231-A/B** | 1 | 1 | 0 | 26 | 0 | 23 | 0 |
| **16061583** | 24 | 24 | 23 | 3 | 23 | 0 | 23 |
| **16091583** | 1 | 1 | 0 | 26 | 0 | 23 | 0 |

**Supplementary Table S4:** SNP difference matrix for samples in cluster 3

|  | 12111121 | 12511121 | 12511122-B | 12881121 | 15171474 | 16021583 | 16041351-B | 16361583-B | 16451233 | 16491231 |
| --- | --- | --- | --- | --- | --- | --- | --- | --- | --- | --- |
| 12111121 | 0 | 0 | 0 | 2 | 1 | 0 | 0 | 1 | 1 | 1 |
| 12511121 | 0 | 0 | 0 | 1 | 0 | 0 | 0 | 0 | 0 | 0 |
| 12511122-B | 0 | 0 | 0 | 2 | 1 | 0 | 0 | 1 | 1 | 1 |
| 12881121 | 2 | 1 | 2 | 0 | 0 | 1 | 2 | 0 | 0 | 1 |
| 15171474 | 1 | 0 | 1 | 0 | 0 | 1 | 1 | 0 | 0 | 0 |
| 16021583 | 0 | 0 | 0 | 1 | 1 | 0 | 0 | 1 | 1 | 1 |
| 16041351-B | 0 | 0 | 0 | 2 | 1 | 0 | 0 | 1 | 1 | 1 |
| 16361583-B | 1 | 0 | 1 | 0 | 0 | 1 | 1 | 0 | 0 | 0 |
| 16451233 | 1 | 0 | 1 | 0 | 0 | 1 | 1 | 0 | 0 | 0 |
| 16491231 | 1 | 0 | 1 | 1 | 0 | 1 | 1 | 0 | 0 | 0 |

**Supplementary Figure S5:** Heatmap showing the SNP distance between the non-typhoidal Salmonella samples in cluster 1 (n=7), ranging from 0 to 3.


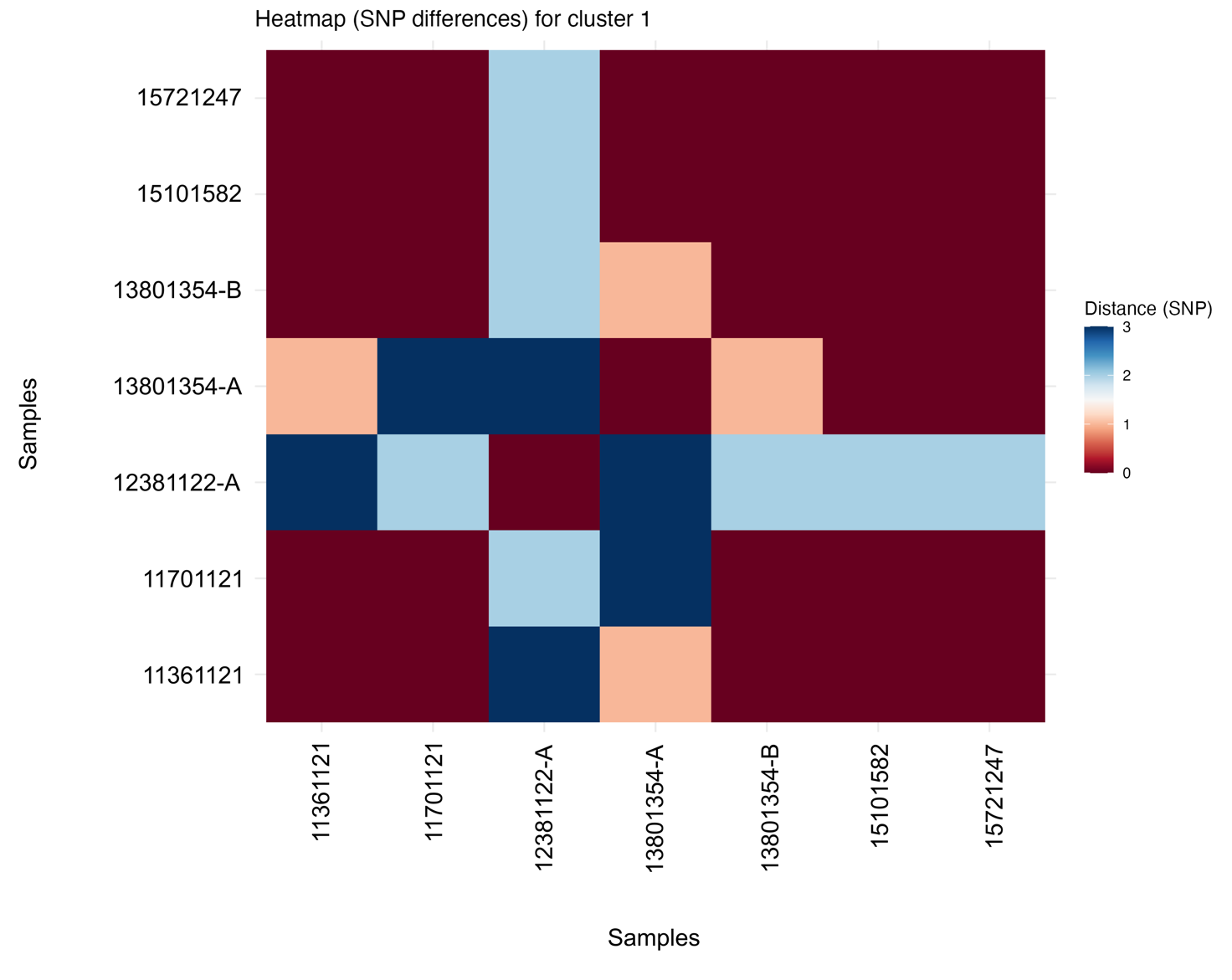


**Supplementary Figure S6:** Heatmap showing the SNP distance between the non-typhoidal Salmonella samples in cluster 2 (n=8), ranging from 0 to 20.


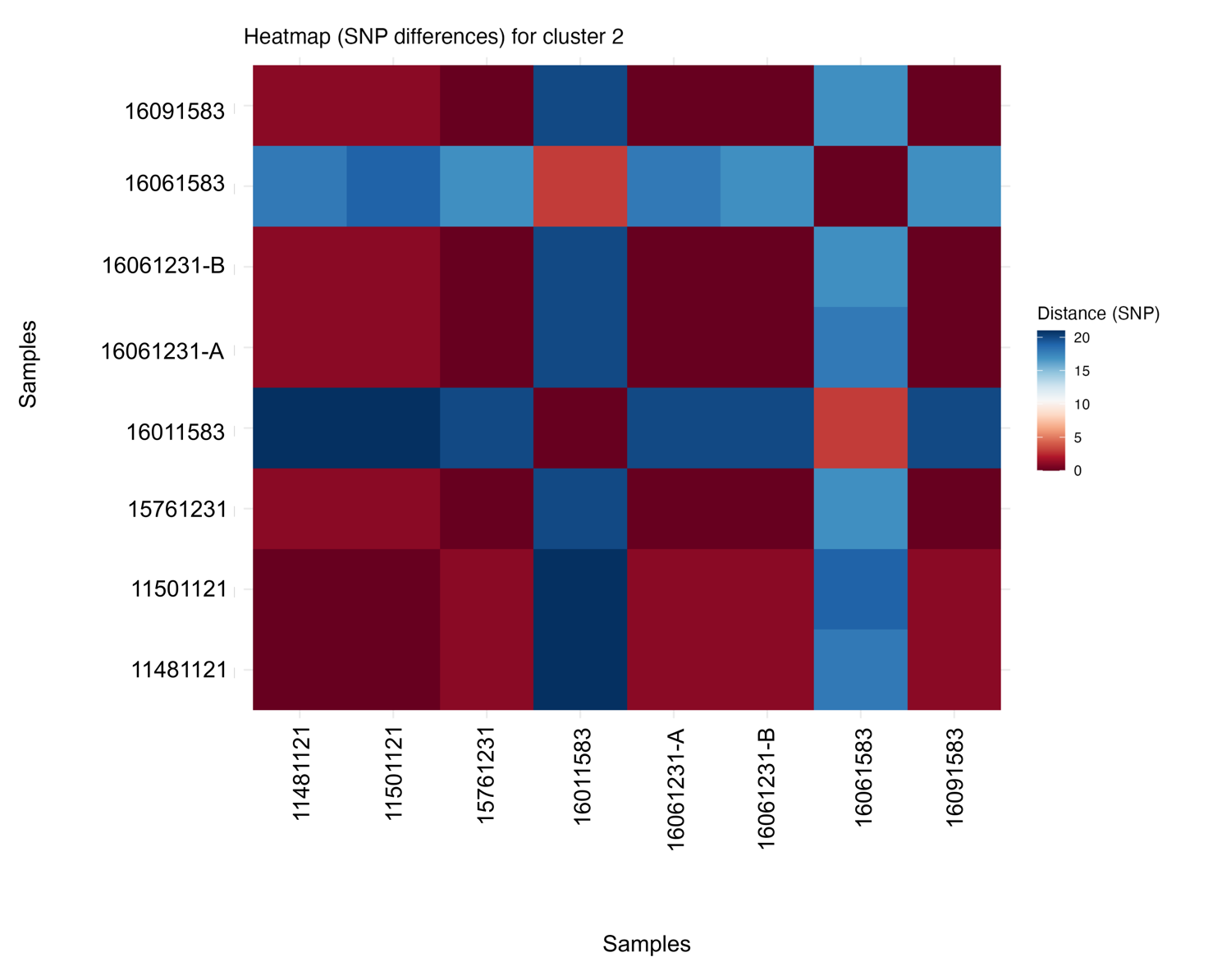


**Supplementary Figure S7:** Heatmap showing the SNP distance between the non-typhoidal Salmonella samples in cluster 3 (n=6), ranging from 0 to 1.


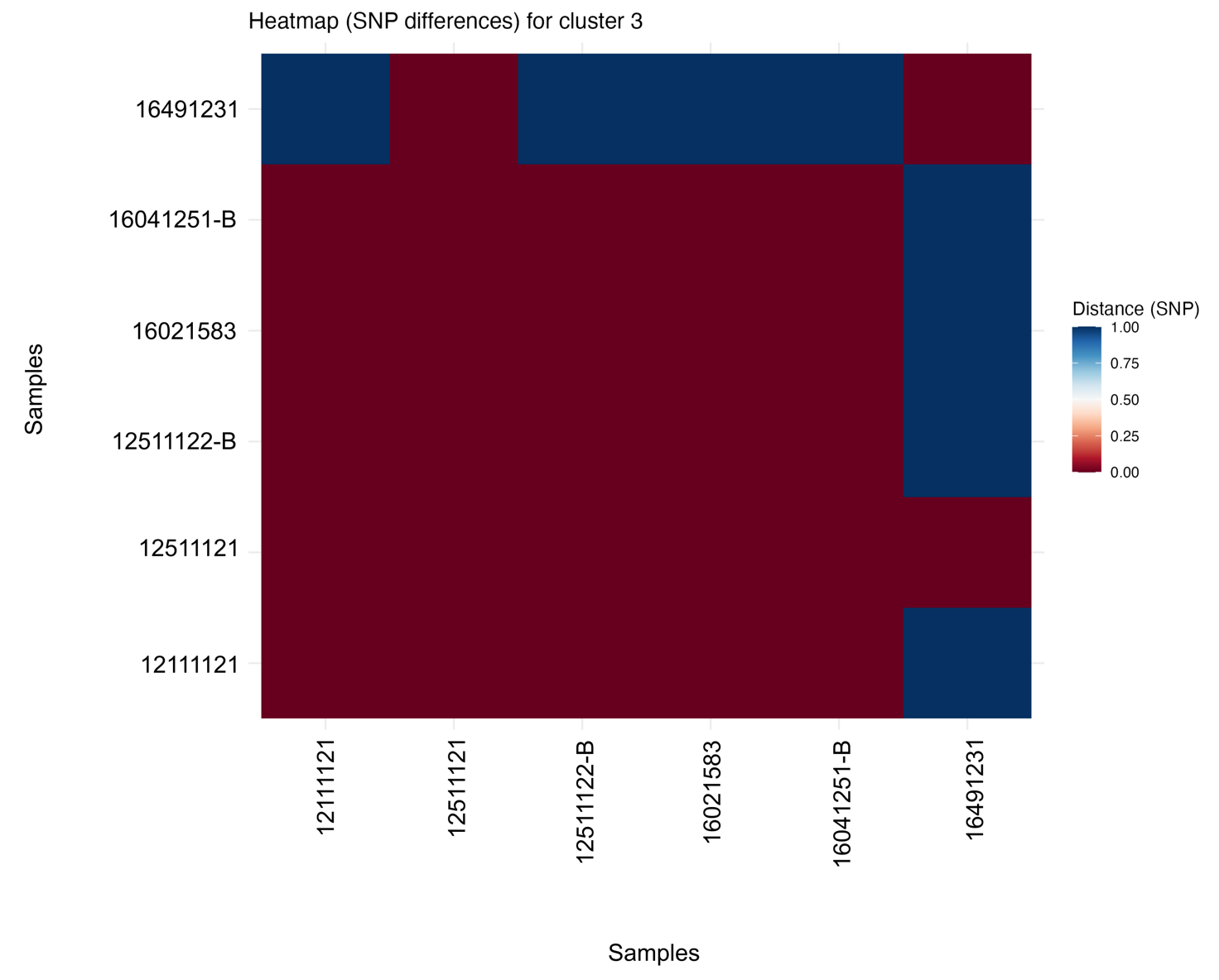

Supplement: Supplementary file 1 — Supplementary Material 1. [file 12879_2024_9800_MOESM1_ESM.docx]
